# Association between Hypothyroidism Onset and Alzheimer Disease Onset in Adults with Down Syndrome

**DOI:** 10.3390/brainsci11091223

**Published:** 2021-09-16

**Authors:** Florence Lai, Nathaniel D. Mercaldo, Cassandra M. Wang, Micaela S. Hersch, Giovi G. Hersch, Herminia Diana Rosas

**Affiliations:** 1 Department of Neurology, Harvard Medical School, Boston, MA 02115, USA; nmercaldo@mgh.harvard.edu (N.D.M.); rosas@helix.mgh.harvard.edu (H.D.R.); 2 Massachusetts General Hospital, Charlestown, MA 02129, USA; 3 McLean Hospital, Belmont, MA 02478, USA; 4 Harvard College, Harvard University, Cambridge, MA 02138, USA; cassandrawang@college.harvard.edu; 5 School of Nursing, Simmons University, Boston, MA 02115, USA; micaela.hersch@simmons.edu; 6 College of Arts & Sciences, Boston University, Boston, MA 02215, USA; herschgi@bu.edu

**Keywords:** Down syndrome, early-onset Alzheimer disease, late-onset Alzheimer disease, hypothyroidism, thyroid autoantibodies, TSH, Free T4, APOE Ɛ4

## Abstract

Adults with Down syndrome (DS) have an exceptionally high frequency of Alzheimer disease (AD) with a wide variability in onset, from 40 to 70 years of age. Equally prevalent in DS is hypothyroidism. In this study, we sought to quantify the relationship between the two. A total of 232 adults with DS and AD were stratified into three AD onset age groups: early (<47 years), typical (48–59), and late (>59). Among patients with available data, differences in the distributions of demographics, hypothyroidism variables (presence, age of onset), thyroid function tests, thyroid autoantibodies, and APOE genotypes were assessed (e.g., chi-squared, Mann–Whitney tests). Spearman and partial Spearman correlations and ordinal logistic regression models were constructed to quantify the association between ages of AD and hypothyroidism onset with and without covariate adjustments. We observed a positive association between the ages of AD and hypothyroidism onset after accounting for APOE-Ɛ4 (correlation: 0.44, 0.24, 0.60; odds ratio: 1.09, 1.05–1.14). However, an early age of hypothyroidism onset and the presence of the APOE-Ɛ4 allele were independently associated with the early age of AD onset. Similar findings were observed when accounting for other factors. Our study provides evidence for the importance of hypothyroidism and associated pathological mechanisms for risk of AD in DS.

## 1. Introduction

Adults with Down syndrome (DS) have an especially high risk for developing Alzheimer disease (AD), with onset at least two decades earlier than in the general population [1,2]. Although there is great variation in the age of AD onset, from as early as 40 to as late as 70 or older [3,4], this variability in onset is poorly understood. The leading hypothesis for the pathogenesis of AD in DS has been attributed to overexpression of the gene for amyloid precursor protein (APP) located on the triplicated chromosome 21 [5], although factors other than amyloid likely influence the wide range of AD onset age in those with DS, just as they do in sporadic AD. Some of these factors include not only the Apolipoprotein Ɛ4 (APOE Ɛ4) genotype, but other genetic factors, as well as environmental or biological factors and co-existing medical conditions. 

Thyroid dysfunction, including congenital, subclinical, and autoimmune thyroid conditions [6], is a very common medical co-morbidity in individuals with DS. In a large meta-analysis of over 6000 children and adults with DS, hypothyroidism was present in almost 40% [7]; similar rates (46%) of hypothyroidism were reported in another study that included only older adults [8]. Congenital hypothyroidism, believed to be primarily due to thyroid hypoplasia [9,10], is estimated to occur in a much higher frequency in children with DS than in the general population [6]. Subclinical hypothyroidism, defined as thyroid-stimulating hormone (TSH) levels above the standardized normal thyroid hormone levels, can occur in approximately one-quarter of those with DS [11]. Autoimmune thyroiditis also occurs with a high frequency in DS; autoantibodies against the thyroid such as thyroid peroxidase (TPO) antibodies are present in almost one-third of those with DS [12], with a high likelihood of conversion to overt hypothyroidism [11].

Thyroid disorders have been recognized as important risk factors in sporadic AD [13,14,15]. Thyroid hormones play a critical role in cognition throughout life, beginning in infancy, and deficiency has been associated with impaired cognition in memory and learning [16], similar to the cognitive impairments in AD. Indeed, hypothyroidism has been recognized as a “reversible dementia” [17], such that the standard of care in the evaluation of individuals with dementia includes screening for thyroid disorders. Both hypothyroidism and AD increase with age [18,19], and the presence of hypothyroidism has been associated with an increased risk for AD [20,21], although no direct cause–effect relationship has been established [22].

Although there is abundant evidence for the significant prevalence of hypothyroidism and AD in adults with DS [1,8,23,24,25], to the best of our knowledge, no prior studies have explored the possible connection between the two. Therefore, in this study, we sought to evaluate the association between a history and age of onset of hypothyroidism and the age of onset of AD in a large cohort of adults with DS. 

## 2. Materials and Methods

IRB approval for a medical records review was obtained from Massachusetts General Hospital. We performed a retrospective study of medical records, including comprehensive medical and neurological history and cognitive assessments, of adults with Down syndrome who had been followed prospectively on an annual basis in a neurology DS subspecialty clinic. A total of 232 patients were identified who had been diagnosed with possible or probable AD, based on the criteria developed by the AAMR-IASSID Working Group for the Establishment of Criteria for the Diagnosis of Dementia in Individuals with Developmental Disability [26]. Patients were classified into two groups of premorbid level of intellectual disability (LID) based on IQ scores or functional ability: (1) mild/moderate LID: IQ between 40 and 70, ability to perform most activities of daily living (ADL), and reasonable language skills; (2) severe/profound LID: IQ < 40, needing at least some assistance in ADLs, with limited language skills. 

### 2.1. Clinical Assessments

Patients underwent annual clinical evaluations and cognitive assessments, including tests standardized for use in individuals with DS (Test of Severe Impairment [27], Verbal Fluency Test [28] and the Dementia Questionnaire for People with Learning Disabilities [26]). Dementia status was determined by a neurologist with experience in diagnosing AD in the DS population. Blood collected at the time of clinical diagnosis of AD included thyroid function tests (TSH and Free T4), Vitamin B12 levels, and APOE genotyping; in a subset of the cohort, blood was sent for the evaluation of thyroid autoantibodies including TPO antibodies and/or thyroglobulin (Tg).

Age of onset of AD was determined based on at least a one-year progressive decline in two or more cognitive domains. Early onset was defined as having an AD onset greater than one standard deviation (SD) below the mean age of AD onset for the cohort, and late onset was defined as greater than one SD above the mean age of AD onset for this group. In our cohort, the mean (SD) age of AD onset was 53 (6). Therefore, early AD onset refers to onset before the age 47, typical AD onset as occurring between ages 47 and 59, and late AD onset as occurring after the age of 59.

Age of hypothyroidism onset, which was obtained from caregivers and available for 111 of the 152 patients (73%) with a reported history of hypothyroidism, was the age at which thyroid supplementation was first started based on abnormal thyroid function tests. 

### 2.2. Statistical Analyses

Descriptive summaries were computed by age of AD diagnosis (early, typical, or late). Continuous variables were summarized using either the mean/standard deviation (SD) or using the median and interquartile range (IQR, 25th–75th percentile). If missing data were observed, the frequency of non-missing variable responses was augmented to the summaries of the continuous variables (e.g., mean (SD); n or median (IQR); n). Categorical variables were summarized as percentages and frequencies of non-missing responses. Using a complete-case analysis, preliminary differences in the distributions of categorical and continuous variables by diagnosis were assessed using either the chi-squared test/Fisher’s exact test or the Mann–Whitney test, respectively. The Spearman correlation coefficient and its 95% confidence interval (CI) were computed to summarize the monotonic relationship between the ages of hypothyroidism and AD onset. 

Partial Spearman correlation coefficients were also computed between ages of hypothyroidism onset and age of AD onset, while separately accounting for each demographic variable or co-varying medical conditions of interest (sex, APOE Ɛ4 status, body mass index (BMI), history of vitamin B12 deficiency, history of obstructive sleep apnea (OSA), and level of intellectual disability). An exploratory series of proportional odds logistic regression models were constructed to quantify the association between the age of AD onset and age of hypothyroidism onset while separately accounting for each demographic or covarying medical condition and their interactions. Wald tests were performed to assess model complexity (non-linear terms, interaction effects). Parameter estimates, 95% CI, and *p*-values were computed to summarize the final regression models.

## 3. Results 

### 3.1. Demographics

Demographics for the cohort and for each diagnostic group are provided in Table 1. The cohort included 36 individuals with early AD onset, 160 with a typical age of AD onset and 36 with late AD onset. Differences in the distribution of sex and the level of intellectual disability were not observed across the three age of AD onset groups (*p* = 0.776 and *p* = 0.265, respectively). There was a significantly higher frequency of patients carrying an APOE Ɛ4 allele (Ɛ3/4 and Ɛ4/4) in the early AD onset group than in the later AD onset groups (*p* = 0.040).

A history of hypothyroidism was present in 58.3% of the early, 65.6% of the typical and 72.2% of the late AD onset cases; there was no significant difference in the frequency of hypothyroidism amongst groups (*p* = 0.463). There was no significant difference in BMI at the time of AD diagnosis (*p* = 0.970). There was, however, a higher prevalence of a diagnosis of OSA in the early AD onset group (*p* = 0.048).

Given previous reports of the known relationship between thyroid dysfunction and cognitive impairment [16,29], we sought to confirm that individuals were euthyroid at the time of AD diagnosis (Table 2). Among patients with a measured TSH value, we were unable to detect differences in the distribution of these values by age of AD onset (continuous: *p* = 0.616, categorized TSH: *p* = 0.310). Approximately 3% of the early, 7% of the typical, and 9% of the late AD onset groups had TSH levels less than 0.34 uIU/mL, suggesting a possible hyperthyroid state. Approximately 3% of the early, 15% of the typical, and 15% of the late AD onset patients had TSH levels greater than 5 ulU/mL, suggesting that they may have been inadequately treated at the time of AD diagnosis. Differences in the distributions of Free T4 levels were not detected amongst the three groups (*p* = 0.277).

### 3.2. Association between the Age of Onset of AD and Age of Onset of Hypothyroidism

Among individuals with a history of hypothyroidism and a reported age of hypothyroidism onset, we observed a significant difference in the age of hypothyroidism by the age of AD onset (*p* = 0.003; Table 1). More specifically, individuals belonging to the early AD onset group had a significantly earlier age of a diagnosis of hypothyroidism, followed by the typical onset, and then by the late AD onset group. Importantly, at the time of evaluation for AD, the majority of the cohort was euthyroid; there was no significant difference amongst the cohorts with respect to TSH or Free T_4_ blood concentrations. 

We subsequently evaluated the association between the age of hypothyroidism onset and age of AD onset. The Spearman correlation between the age of hypothyroidism onset and age of AD onset was 0.43 (95% CI: 0.27, 0.57) (*p* < 0.001). When assuming a linear representation of age of hypothyroidism (*p*_anova_ > 0.05), we observed that for each year increase in the age of hypothyroidism onset, the unadjusted odds of having a later age of AD onset increased by a factor of 1.09 (95% CI: 1.05–1.12). Thus, among patients with hypothyroidism, those who developed it earlier appeared to have an earlier age of AD onset. 

Similar findings were observed after accounting for each demographic variable and co-varying medical condition of interest (history of vitamin B12 deficiency: 0.44 (0.25, 0.59); history of OSA: 0.45 (0.26, 0.60); BMI: 0.44 (0.22, 0.62); APOE Ɛ4 status: 0.44 (0.24, 0.60), sex: 0.43 (0.24, 0.58); level of intellectual disability: 0.46 (0.28, 0.61); all *p* < 0.001). There was insufficient evidence to conclude that a non-linear coding of age of hypothyroidism onset and the interaction between age of hypothyroidism onset and each demographic variable improved the model fit of age of AD onset (all *p*_anova_ > 0.05). Thus, we observed that for each year increase in the age of hypothyroidism onset, the adjusted odds of having a later age of AD onset increased by a factor of: 1.09 (95% CI: 1.05–1.12) after adjusting for history of vitamin B12 deficiency, 1.08 (1.05, 1.12) after adjusting for sex, 1.09 (1.05, 1.13) after adjusting for history of OSA, 1.10 (1.05, 1.14) after adjusting for BMI, 1.10 (1.06, 1.14) after adjusting for level of intellectual disability, and 1.09 (1.05, 1.14) after adjusting for APOE Ɛ4 status. 

### 3.3. Thyroid Autoantibodies

To evaluate the potential contribution of autoimmune thyroiditis to the risk of developing AD early, we evaluated TPO antibody levels; these were available for only approximately 41% (96/232) of the entire cohort (Table 3). Among those with a recorded TPO value, we were unable to detect differences in the distribution of TPO values by the age of AD onset (*p* = 0.591). The frequency of an elevated TPO, defined as a level above 9 IU/mL, was present in approximately 33% of the early, 40% of the typical, and 32% of the late AD onset group (*p* = 0.731). 

Thyroglobulin (Tg) antibody levels were available for approximately 32% (74/232) of the cohort. Among those with a recorded Tg value, values tended to be less than 4 IU/mL, but we were unable to detect differences in the distribution of these values by the age of AD onset (*p* = 0.533).

## 4. Discussion

It is well known that both hypothyroidism and AD occur in high frequency in those with DS; however, our study is the first to explore the potential relationship between the age of onset of hypothyroidism and age of onset of AD in DS. Although an earlier age of AD onset in the DS population has been reported to occur, on average, 20 years earlier than in the neurotypical population, it has generally been ascribed to the triplication of APP on chromosome 21. However, our data suggest that the presence of hypothyroidism early in life may also provide its own contribution to risk for AD in DS. This was true even when other co-variates and other medical co-morbidities known to independently contribute to cognitive dysfunction were taken into consideration. Earlier onset of hypothyroidism within the DS population was associated with an even greater risk for the earlier onset of AD. Although the biological mechanisms that might explain this are as yet unknown, one possibility is that thyroid hormone itself may impact the expression of amyloid precursor protein, such that reduced thyroid hormone levels increase APP expression and elevations of pathogenic amyloid [30]. This effect may be even more significant in those with DS who have a much higher amyloid burden. 

As expected, the presence of an APOE Ɛ4 allele was associated with an earlier onset of AD in DS, as has been reported by others [31]; however, we did not detect an interaction between the presence of the APOE Ɛ4 allele and the relationship between the age of onset of AD and age of hypothyroidism onset. Our findings suggest that APOE Ɛ4 and early age of hypothyroidism may each contribute independently to the risk for early AD onset in the DS population. 

In a subset of our cohort for whom results for serum thyroid autoantibodies were available, approximately 40% had thyroid autoantibodies as evidence of autoimmune thyroiditis, consistent with what has been reported previously in DS [32]. A similar frequency of thyroid autoantibodies was present in each of the three age of AD onset groups, suggesting that the presence of autoantibodies did not confer added risk for the development of early AD in the DS population. However, we did not have sufficient sample size to evaluate the potential interaction with the presence of thyroid autoantibodies. Nevertheless, given the comparable distribution and similar blood levels across the three groups, it appears unlikely that thyroid autoantibodies confer added risk for early AD onset in the DS population. Future studies are needed to fully evaluate the potential contribution of autoimmune thyroiditis to AD risk in DS. 

It is important to point out that at the time of an AD diagnosis, a majority of patients in each of the cohorts had TSH levels within the normal ranges, confirming that the majority were euthyroid at the time of AD diagnosis, and that there were no significant differences amongst TSH levels across the groups. Similarly, the overall frequency of hypothyroidism in our sample was consistent with the findings in a large meta-analysis of adults with DS [8], indicating that our cohort is representative of the DS population at large. A diagnosis of hypothyroidism appeared to occur, on average, more than a decade prior to a diagnosis of AD (as defined as the difference between the age of AD onset and age of hypothyroidism onset), irrespective of age of AD onset. Alterations in thyroid function have been associated with a higher risk of developing AD later in life in the neurotypical population [33], suggesting that complex interactions between thyroid hormone, thyroid function and risk for AD may be particularly salient in the DS population. 

## 5. Limitations 

The primary limitations of this study include: (1) the retrospective nature of the study design; (2) the possible measurement error associated with ages of AD onset and hypothyroidism onset; and (3) the degree of missingness especially involving the thyroid autoantibody panels. The validity of these results assumes that the covariate missingness mechanism is completely random. Nevertheless, the results of this study need to be studied further in larger (and ideally prospectively collected) datasets. 

## 6. Conclusions

In conclusion, our study suggests that the early onset of hypothyroidism in DS is significantly associated with an early onset age of AD, and that it is independent of APOE Ɛ4 allele status, BMI, vitamin B12 status, or presence of OSA. This emphasizes the importance of early testing for TSH, thyroid hormone and thyroid autoantibodies, and treatment with thyroid replacement as needed. Future studies are needed to determine the mechanisms by which a history of hypothyroidism affects AD risk and onset, including its relationship with other genetic, inflammatory or metabolomic alterations present in adults with DS. 

## Figures and Tables

**Table 1:** Demographic summaries by age of AD onset group.

	Early	Typical	Late	*p*
	N = 36	N = 160	N = 36	
AD onset (years)				<0.001
Mean (SD)	43.81 (1.92)	52.92 (3.72)	62.19 (2.30)	
Sex, % (n)				0.776
Male	61.1 (22)	55.0 (88)	58.3 (21)	
Female	38.9 (14)	45.0 (72)	41.7 (15)	
Level of Intellectual Disability, % (n)				0.265
Mild/Moderate	72.2 (26)	62.8 (98)	53.1 (17)	
Profound/Severe	27.8 (10)	37.2 (58)	46.9 (15)	
APOE Ɛ4 allele, % (n)				0.04
Absent	62.1 (18)	77.0 (107)	90.0 (27)	
Present (3/4,4/4)	37.9 (11)	23.0 (32)	10.0 (3)	
BMI (kg/m^2^)				0.97
Mean (SD); n	30.19 (5.06); 26	29.93 (6.34); 123	30.14 (4.67); 28	
History of hypothyroidism, % (n)				0.463
No	41.7 (15)	34.4 (55)	27.8 (10)	
Yes	58.3 (21)	65.6 (105)	72.2 (26)	
Hypothyroidism onset (years)				0.003
Mean (SD); n	34.1 (10.86); 17	42.2 (9.57); 78	45.7 (11.40); 16	
History of obstructive sleep apnea, % (n)				0.048
Absent	61.1 (22)	75.4 (120)	86.1 (31)	
Present	38.9 (14)	24.5 (39)	13.9 (5)	
History of vitamin B12 deficiency, % (n)				0.993
Absent	80.6 (29)	79.9 (127)	80.6 (29)	
Present	19.4 (7)	20.1 (32)	19.4 (7)	

**Table 2:** Thyroid function tests: summaries by age of AD onset group.

	Early	Typical	Late	*p*
	N = 36	N = 160	N = 36	
TSH (ulU/mL, continuous)				0.616
Median (IQR); n	2.46 (1.78, 3.04); 33	2.04 (1.23, 3.63); 148	2.10 (1.18, 3.37); 34	
TSH (ulU/mL, categorical), % (n)				0.31
0.00–0.33	3.0 (1)	6.8 (10)	8.8 (3)	
0.34–5.00	93.9 (31)	78.4 (116)	76.5 (26)	
>5.00	3.0 (1)	14.9 (22)	14.7 (5)	
Free T4 (ng/dL, continuous)				0.277
Median (IQR); n	0.90 (0.83, 1.08); 18	1.00 (0.90, 1.20); 95	0.95 (0.90, 1.30); 28	
Free T4 (ng/dL, categorical), % (n)				0.832
0.00–0.89	27.8 (5)	20.0 (19)	21.4 (6)	
0.90–1.90	72.2 (13)	77.9 (74)	78.6 (22)	
>1.90	0.0 (0)	2.1 (2)	0.0 (0)	

**Table 3:** Thyroid antibody summaries by age of AD onset group.

	Early	Typical	Late	*p*
	N = 36	N = 160	N = 36	
TPO (IU/mL, continuous)				0.591
Median (IQR); n	1.30 (1.10, 16.50); 9	3.75 (1.40, 24.62); 64	4.01 (1.53, 10.30); 22	
TPO (IU/mL, categorical), % (n)				0.731
0.00–9.00	66.7 (6)	59.4 (38)	68.2 (15)	
>9.00	33.3 (3)	40.6 (26)	31.8 (7)	
Tg (IU/mL, continuous)				0.533
Median (IQR); n	2.65 (1.80, 3.88); 8	1.80 (1.80, 2.45); 50	1.80 (1.80, 1.80); 16	
Tg (IU/mL, categorical), % (n)				0.701
0.00–4.00	75.0 (6)	86.0 (43)	81.2 (13)	
>4.00	25.0 (2)	14.0 (7)	18.8 (3)	

## Data Availability

Data are available for review by request to corresponding author.
